# Supplementary material for: Complexome profiling of the Chlamydomonas psb28 mutant reveals TEF5 as an early PSII assembly factor
Source: Plant Cell. 2025 Mar 17;37(6):koaf055. doi: 10.1093/plcell/koaf055 (PMC12164590; doi:10.1093/plcell/koaf055)
Supplement: koaf055_Supplementary_Data [file koaf055_supplementary_data.zip › psb28_interactive.zip!/plots/intensity/qualityPlots/peptides/LHCA7_Cre16.g687900.t1.2.html]

### Cre16.g687900.t1.2

Trivial name: LHCA7  
SeqCoverage [median]: 78.80  
Mass: 26.22 kDa  
  
 

| Sequence | MissedCleavage | Mass [Da] | wtIntensity | muIntensity | Score |
| --- | --- | --- | --- | --- | --- |
| FAMLGAAGIILTSIGAK | 0 | 1632.93 | 509808.06 | 430530.13 | 224.66 |
| FFDPMGLSR | 0 | 1068.51 | 1958690.00 | 3238268.41 | 140.35 |
| FFDPMGLSRGDAAK | 1 | 1510.72 | 55781.84 | 131318.85 | 101.32 |
| GDAAKYQEYK | 1 | 1171.55 | 8560.25 | 16582.49 | 112.13 |
| GLENGYPGGR | 0 | 1018.48 | 442.32 | 2888.52 | 174.16 |
| GPLDNLADHLADPNHVNFATNGVSIPIA | 0 | 2881.44 | 0.00 | 75424.63 | 130.65 |
| NNIDFPTLMVIQFYLMGWAETKR | 1 | 2786.39 | 2639.54 | 0.00 | 59.94 |
| NPGSQADGSFLGFTEEFK | 0 | 1929.87 | 157337.29 | 32162.38 | 365.28 |
| NPGSQADGSFLGFTEEFKGLENGYPGGR | 1 | 2930.35 | 75895.14 | 0.00 | 169.60 |
| PVWFPGNPPPAHLDGSLAGDYGFDPLFLGQEPQTLK | 0 | 3876.92 | 4543.29 | 0.00 | 63.85 |
| RWYDFKNPGSQADGSFLGFTEEFK | 2 | 2825.31 | 3052.14 | 0.00 | 48.39 |
| VGLGFPEWYDAGK | 0 | 1437.69 | 426958.81 | 140099.25 | 233.14 |
| VGLGFPEWYDAGKVVVEK | 1 | 1992.04 | 76620.52 | 0.00 | 133.78 |
| WYDFKNPGSQADGSFLGFTEEFK | 1 | 2669.21 | 42680.96 | 0.00 | 180.90 |
| WYDFKNPGSQADGSFLGFTEEFKGLENGYPGGR | 2 | 3669.68 | 17244.50 | 0.00 | 120.42 |
| WYVQAELVHGR | 0 | 1356.69 | 766862.91 | 520930.82 | 152.64 |
| YQEYKQKEVK | 2 | 1341.69 | 2029.60 | 3385.81 | 103.91 |
|  |  |  | 4109147.2 | 4591591.3 |  |
